# Sub-nanosecond signal propagation in anisotropy-engineered nanomagnetic logic chains

**DOI:** 10.1038/ncomms7466

**Published:** 2015-03-16

**Authors:** Zheng Gu, Mark E. Nowakowski, David B. Carlton, Ralph Storz, Mi-Young Im, Jeongmin Hong, Weilun Chao, Brian Lambson, Patrick Bennett, Mohmmad T. Alam, Matthew A. Marcus, Andrew Doran, Anthony Young, Andreas Scholl, Peter Fischer, Jeffrey Bokor

**Affiliations:** 1 Department of Electrical Engineering and Computer Sciences, University of California, Berkeley, California 94720, USA; 2 Intel Corp., 2200 Mission College Boulevard, Santa Clara, California 95054, USA; 3 Thorlabs Inc., 56 Sparta Avenue, Newton, New Jersey 07860, USA; 4 Center for X-ray Optics, Lawrence Berkeley National Laboratory, Berkeley, California 94720, USA; 5 Daegu Gyeongbuk Institute of Science and Technology, Daegu 711-873, Korea; 6 iRunway, 2906 Stender Way, Santa Clara, California 95054, USA; 7 Intel Corp., 5200 NE Elam Young Parkway, Hillsboro, Oregon 97124, USA; 8 Advanced Light Source, Lawrence Berkeley National Laboratory, Berkeley, California 94720, USA; 9 Department of Physics, University of California, Santa Cruz, California 94056, USA

## Abstract

Energy efficient nanomagnetic logic (NML) computing architectures propagate binary information by relying on dipolar field coupling to reorient closely spaced nanoscale magnets. Signal propagation in nanomagnet chains has been previously characterized by static magnetic imaging experiments; however, the mechanisms that determine the final state and their reproducibility over millions of cycles in high-speed operation have yet to be experimentally investigated. Here we present a study of NML operation in a high-speed regime. We perform direct imaging of digital signal propagation in permalloy nanomagnet chains with varying degrees of shape-engineered biaxial anisotropy using full-field magnetic X-ray transmission microscopy and time-resolved photoemission electron microscopy after applying nanosecond magnetic field pulses. An intrinsic switching time of 100 ps per magnet is observed. These experiments, and accompanying macrospin and micromagnetic simulations, reveal the underlying physics of NML architectures repetitively operated on nanosecond timescales and identify relevant engineering parameters to optimize performance and reliability.

## Abstract (web summary)

Closely-spaced anisotropically-engineered single-domain nanomagnets may be exploited to encode and transmit binary information. Here, Gu *et al*. use time-resolved X-ray microscopy to image signal propagation at the intrinsic nanomagnetic switching limit in permalloy nanomagnet chains.

## Graphical abstract

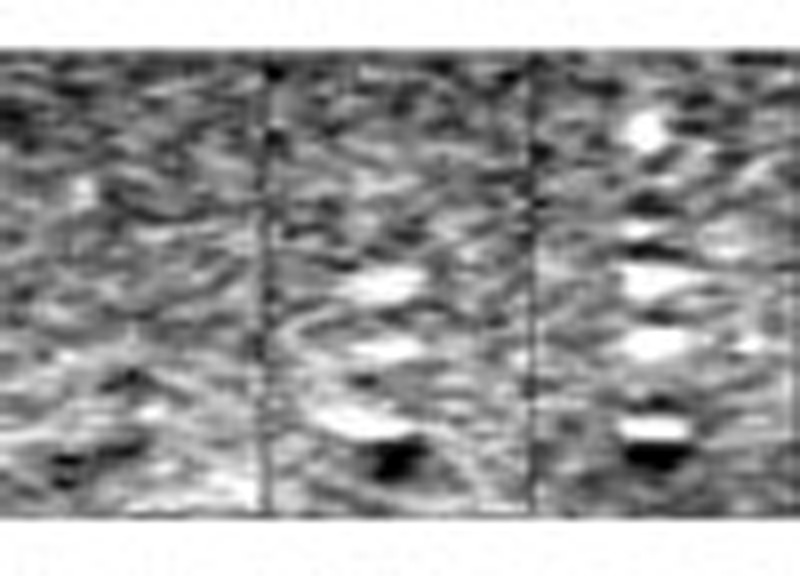

Nanomagnetic logic (NML) is a computational architecture that promises ultralow energy dissipation per operation^1^,^2^,^3^,^4^. In NML, the magnetization of single-domain ferromagnetic thin-film islands are coupled by dipolar fields generated from adjacent islands. Single islands are typically ellipse or rectangular-shaped; this confines the magnetic easy axis to the major axis. When islands are arranged in a line parallel to the minor axis (magnetic hard axis), the nearest-neighbour magnetic dipolar field coupling imparts a preference for the magnetization of these neighbouring islands to align antiparallel, forming a spatial logical inverter^5^. An extended series of these closely spaced nanomagnets, called a chain, propagates binary information from one end to the other sequentially through a series of inversions, performing a function similar to conventional integrated circuit interconnects but with potentially lower dissipation energy per switching event^2^,^6^. These chains are a fundamental building block of NML architectures. To perform a logic operation, the magnetization state of an output magnet is determined by a majority vote of the magnetic state of three input magnets that surround the output; this majority logic gate has been experimentally demonstrated^2^ and is projected to switch at energies near 1 eV (0.16 aJ), making NML a candidate for computing with energy dissipation approaching the fundamental thermodynamic limit^7^.

To perform multiple, successive logic operations, the entire NML architecture (chains and majority logic gates) must be reinitialized after each operation. This resetting process is known as clocking, and in this work we use pulsed nanosecond on-chip magnetic fields^8^ to drive the magnetization of all nanomagnets in a chain to saturation along their magnetic hard axes. This places each magnet in an energetically unstable (null) state that, upon removal of the clock field, becomes coupled to a nearest-neighbour magnet by the dipolar fields. The time-dependent relaxation from the null state in rectangular or elliptically shaped magnets, however, can also be affected by factors such as thermal fluctuations^9^,^10^,^11^,^12^,^13^ and non-ideal magnetic anisotropies^14^. These aberrations can drive the magnetization of individual islands to spontaneously switch out of sequence, forming an error that spoils the sequentially directed dipolar coupling, which correctly propagates the input information. Error rates have been predicted to increase as a function of shorter clocking pulse lengths approaching nanosecond timescales in chains of five or more ellipse magnets^9^ due to the non-deterministic settling of magnets far from the input. This has motivated efforts to prevent error nucleation in longer chains^10^,^11^,^12^,^13^.

One method to increase the stability of the null state is to artificially engineer a metastable potential well along the hard axis by introducing a biaxial anisotropy component to each individual nanomagnet^11^. Two distinct methods can be used to incorporate this biaxial anisotropy: choosing a material with an intrinsic magnetocrystalline biaxial anisotropy^10^,^11^ or fabricating lithographically-defined notches on both ends of each nanomagnet major axis^15^,^16^,^17^,^18^; the latter option provides an opportunity to controllably tune the relative strength of the biaxial anisotropy. NML chains composed of lithographically-notched nanomagnets have been previously fabricated and were shown to reduce the influence of thermal fluctuations and random lithographic variations to an extent that error-free signal propagation along a chain of eight nanomagnets was observed, driven by a quasi-static, adiabatic clocking process^13^. For NML to be a viable alternative logic architecture, fast, error-free operations, with speeds limited solely by the intrinsic magnetic relaxation time (order 100s of picoseconds), must be repeatable and reliable over successive clocking cycles^19^,^20^,^21^,^22^. Even though reliable high-speed operation has been predicted^11^,^12^,^13^, it has yet to be experimentally studied in any NML architecture.

Previous studies of NML have incorporated static magnetic imaging after clocking chains and assumed corrected signal propagation based on the final observed output state^2^,^5^,^8^,^12^,^13^,^14^,^20^,^22^,^23^,^24^. In this work, we use an X-ray-based imaging technique with 100 ps resolution to directly observe the signal propagation dynamics in chains immediately after clocking. This technique introduces a new method to assess NML reliability on fast timescales and provides a powerful tool to identify previously undetectable signal propagation errors. This new information can help optimize chain engineering to improve reliability for successful and repeatable signal propagation with a fast cycling protocol. First, using magnetic full-field transmission soft X-ray microscopy (MTXM)^25^ at the Advanced Light Source (ALS) synchrtron at Lawrence Berkeley National Laboratory, we statistically analyse the signal propagation reliability of NML chains composed of nanomagnets with lithographically-defined notches, clocked by single nanosecond pulses, with varying magnet dimensions to identify regions of optimal signal propagation and verify that fast clocking can be reliably used. Next, we directly image signal propagation dynamics in a chain of anisotropy-engineered nanomagnets with 100 ps time resolution using magnetic time-resolved photoemission electron microscopy (TR-PEEM)^26^,^27^,^28^ by clocking in sync with pulsed X-rays at a repetition rate of 3 MHz. Finally, we compare both experimental results with computational simulations. With a micromagnetic simulator, we examine the effectiveness and clocking behaviour of nanosecond magnetic field pulses. In addition, we use macrospin simulations to examine relevant experimental parameters which affect the reliability and speed of signal propagation; these include temperature, nanomagnet dimensions and the dipolar coupling strength. These simulations both validate our experimental results and suggest approaches for further technological improvement.

## Results

### Static magnetic imaging and micromagnetic simulations

**Figure 1:**
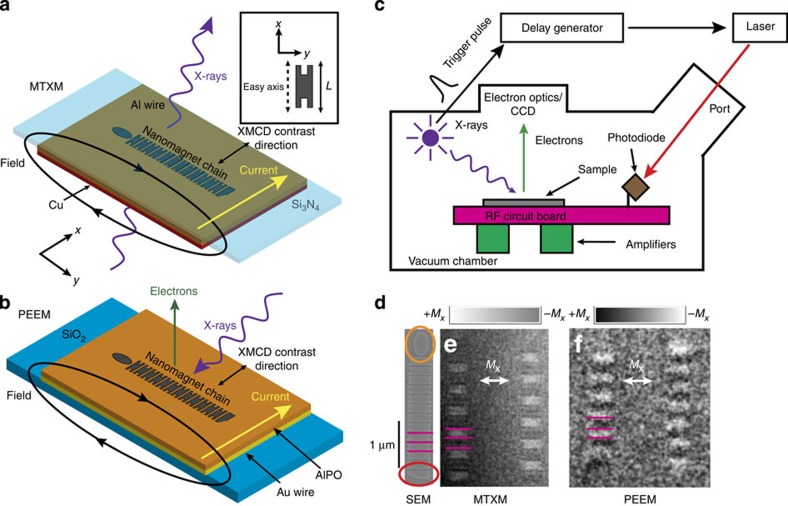
Sample design and X-ray microscopy experimental setup. (**a**,**b**) Schematic of soft X-ray transmission (MXTM) and PEEM experiments, respectively. In both experiments, nanomagnet chains are fabricated on metal wires. Current pulses generating on-chip fields (along *ŷ*) reset the orientation of all nanomagnets after each clocking cycle. XMCD contrast is measured parallel to the nanomagnet easy axes (along 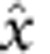) Inset: orientation of the easy axis (along 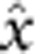) for a nanomagnet of length, *L*, with engineered biaxial stability. (**c**) Schematic of TR-PEEM setup. A trigger pulse generated by the ALS synchrotron excites laser pulses that are focused through a window port of the vacuum chamber onto the photodiode of a customized circuit board (shown in pink) on which the sample (shown in grey) is mounted inside the PEEM vacuum chamber. The circuit contains a series of RF amplifiers (green boxes) that amplify the current pulses from the photodiode to generate on-chip clocking fields, which are synchronized with X-ray pulses by a delay generator. (**d**) Scanning electron microscopy (SEM) of a NML chain with 12 nanomagnets. The input and terminating block magnets are circled in red and orange, respectively. (**e**,**f**) Magnetic contrast images of chains with 12 nanomagnets observed by MTXM and PEEM. The colour scales represent easy axis magnetization. Pink lines distinguish individual nanomagnets in a chain.

To study propagation reliability using MTXM, notched (Fig. 1, inset) nanomagnet chains (Fig. 1d) composed of 12 individual nanomagnets (150 nm wide and of varying length *L* (Fig. 1, inset)) separated by 30 nm, are fabricated along an Al wire on X-ray transparent Si_3_N_4_ membranes of 100 nm thickness by a combination of electron beam and optical lithography, evaporation, liftoff and wet-etching techniques (see Methods section). During the measurement, manually triggered clocking pulses of 3 ns duration generating an 84-mT on-chip field (along *ŷ* as shown in Fig. 1a) reset the chains (see Methods section). Dipolar signal propagation is initialized at the chain input^23^ by magnets with shape anisotropy (indicated by the red oval in Fig. 1d and Supplementary Fig. 1) designed to spontaneously and consistently orient along one direction of the easy axis (along 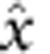 as shown in the inset of Fig. 1) after clocking (Supplementary Note 1). For each length *L*, two nominally identical chains are fabricated with oppositely oriented input magnets (Fig. 1e,f). In addition, an ellipse-shaped block magnet (indicated by the orange oval in Fig. 1d and Supplementary Fig. 1) terminates each chain to stabilize the final nanomagnet after successful signal propagation^8^ (Supplementary Note 1). Static magnetic contrast images taken after triggering a single clock pulse are detected by X-ray magnetic circular dichroism (XMCD)^29^ (see Methods section), which is sensitive to the proportion of the magnetization vector parallel to the propagation direction of the incident X-rays (along 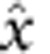 as shown in Fig. 1a). For MTXM, the contrast is obtained by subtracting an image of the final state of the chain from a reference image of the chain magnetically saturated along its easy axis. The magnetization along the easy axis (*M*_*x*_ in the inset of Fig. 1) of magnets that have oriented antiparallel (+*M*_*x*_) to the reference image after the clocking pulse contrast from magnets that have reoriented along the same direction (−*M*_*x*_) as the reference image as shown in Fig. 1e.

**Figure 2:**
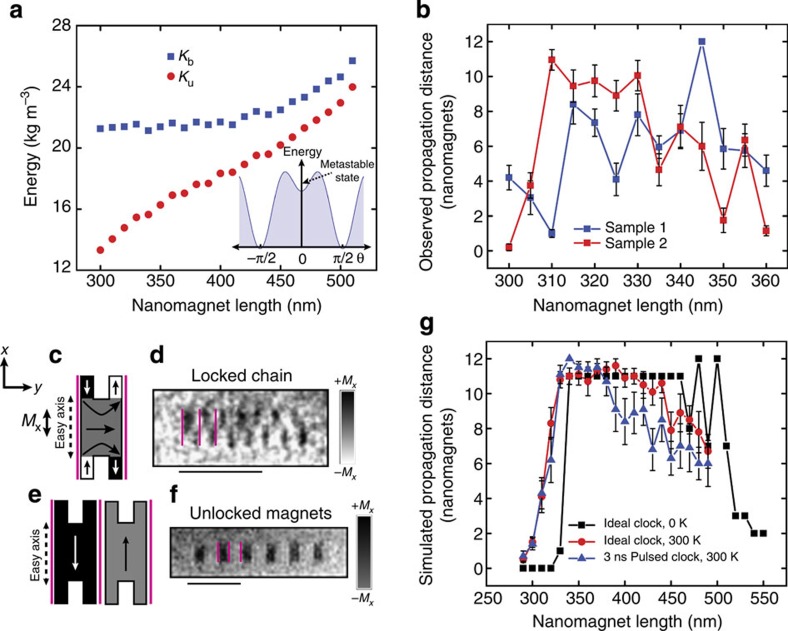
Identifying regions of optimal NML signal propagation for pulsed clocking fields. (**a**) The uniaxial (*K*_u_) and biaxially (*K*_b_) anisotropy energy (red circles and blue squares, respectively) as a function of nanomagnet length for 150-nm-wide notched nanomagnets calculated from an analytical model and OOMMF simulations. Inset: schematic energy diagram with respect to the magnetic orientation from the hard axis (*θ*=0), indicating the biaxial anisotropy-generated metastable state. (**b**) Average signal propagation distance in nanomagnet chains from the input magnet as a function of the nanomagnet length experimentally observed in two samples (red and blue squares) with MTXM. Error bars report the standard error of the mean. (**c**,**d**) Illustration and MTXM image of a chain of seven nanomagnets with their magnetization locked along the hard axis (along *ŷ*) after initialization. Pink lines distinguish individual nanomagnets in the chain. MTXM can resolve the antiparallel domains (parallel to the easy axis along 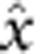) in the notched region of the biaxially engineered nanomagnets. (**e**,**f**) Illustration and MTXM image of perfect signal propagation in a chain of 12 nanomagnets. Pink lines distinguish individual nanomagnets in the chain. The magnetization along the easy axis of neighbouring nanomagnets is oriented antiparallel along 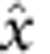. In **d** and **f**, colour scales represent easy axis magnetization, and scale bars represent 1 μm. (**g**) Signal propagation distance of nanomagnet chains from the input magnet as a function of nanomagnet length calculated from OOMMF simulations for ideally initialized chains clocked at 0 K (black squares), 300 K (red circles) and chains initialized by a 3-ns clocking pulse at 300 K (blue triangles). Error bars report the standard error of the mean.

The values of the biaxial anisotropy (*K*_b_) and uniaxial anisotropy (*K*_u_) energies for a single 150-nm-wide notched nanomagnet are calculated as a function of the nanomagnet length *L* in Fig. 2a using an analytical model and object oriented micromagnetic framework (OOMMF)^30^ (Supplementary Note 2) by simulating the dipolar and clocking fields (Supplementary Fig. 2) required to drive the magnetization between the easy and hard axes in a nanomagnet with a lithographically defined anisotropy. The values for *K*_u_ are approximately linear as *L* is varied from 300 to 500 nm, while the fixed notch dimensions for each nanomagnet keep *K*_b_ approximately constant. The ratio of these two energies (*K*_b_:*K*_u_) parameterizes a lithographically defined energy well for the metastable state (Fig. 2a, inset) that the dipolar coupling fields must overcome to correctly reorient initialized magnets. We assess both the clocking stability and signal propagation distance as a function of this ratio.

Figure 2b plots the average experimentally observed signal propagation distance of nanomagnet chains, when clocked as described above, as a function of *L* for two identically processed samples. The propagation distance is defined as the number of nanomagnets (starting at the input) over which the signal propagates correctly before encountering the first error. After propagation is complete, magnets that remain along their hard axes and magnets that are aligned parallel to either neighbour are both considered errors. Owing to the static nature of this measurement, out-of-order switching errors cannot be distinguished. For every value of *L*, four chains were each pulsed 10 times. We observe a systematic propagation distance peak in sample 2 as *K*_u_ is tuned by varying *L*. Error bars represent random variations in signal propagation on different trials. Sample 1 does not clearly show this peak, but rather contains a pair of chains with the same *L* showing perfect signal propagation every trial. Although sample-to-sample process variations lead to widely varying levels of performance, we anticipate that significant improvements in signal propagation distance are possible with more consistent processing.

For nanomagnets with *L* between 300 and 315 nm in Fig. 2b, the energy well set by *K*_b_:*K*_u_ is too deep for the dipolar coupling fields to overcome. After the clocking field is removed, the nanomagnets remain oriented along their hard axes, stabilized by the biaxial anisotropy (that is, trapped, or locked, in the metastable state). Figure 2c depicts the schematic magnetic configuration that is verified by an MTXM image of a chain fully locked in this manner (Fig. 2d). For nanomagnets with *L* between 315 and 360 nm, *K*_b_:*K*_u_ defines a shallower energy well. This reduces the dipolar field coupling required to reorient the magnets; however, this also reduces the stability provided by the metastable state and makes it more susceptible to thermal excitations or anisotropies introduced by processing irregularities. Because of this, we observe reduced signal propagation distances in chains of nanomagnets with *L* >350 nm. The high signal propagation regions in Fig. 2b represent regions in each sample where *K*_b_:*K*_u_ is optimally tuned. Accordingly, Fig. 2e depicts the schematic magnetic configuration and Fig. 2f shows an MTXM image of a chain with perfect signal propagation.

Micromagnetic simulations with OOMMF^30^ provide further insight into the magnetic signal propagation observed in this experiment. In Fig. 2g, we present simulated signal propagation lengths for chains of 12 magnets identical to the experimental dimensions and spacing (bounded by inputs^23^ and blocks^8^; Supplementary Note 1). Initially, we study magnet chains as they evolve from an ideal metastable state (ideal clock). Without thermal fluctuations (0 K, black squares), we observe a relatively large, sharply defined signal propagation region, spanning a range of *L* of over 120 nm in width, of nearly perfect signal propagation. The final magnet in the chain is too strongly coupled to the block and remains oriented in the metastable state. This is an artefact of the simulation and hence a maximum signal propagation of 11 magnets is observed. This block coupling also creates signal propagation noise on the right side of the plot by acting as a nucleation site for errors. In actual NML architectures, we do not anticipate this behaviour. At 300 K (red circles), stochastic thermal effects reduce the high propagation region to ~80 nm wide in *L*. This is because as *K*_b_:*K*_u_ is reduced, thermal energy can assist in prematurely reorienting magnets, creating errors.

Finally, we repeat the same simulations but no longer assume an idealized initialization condition. Instead, the simulated chains are subjected to a trapezoidal-shaped 3-ns-clocked pulse (200 ps rise time and 300 ps fall time), approximating our experimental clock pulse (blue triangles). As with ideal clocking, signal propagation behaviour is similar to idealized conditions and is a function of *K*_b_:*K*_u_, however the short clocking pulse does not provide the same degree of stability when switching to the metastable state. Hence, we find a signal propagation region spanning only 50 nm in *L*. We also observe that the pulsed field, in conjunction with stochastic variability, produces a slightly non-uniform initialized state among individual magnets in a chain. This initialization randomness exacerbates the trend of reduced signal propagation at larger values of *L*, already present in ideally initialized chains. Nevertheless, despite fabrication-related imperfections, we observe a high signal propagation region spanning 20 nm in *L* in one sample (Fig. 2b). The magnitude of this span is the same order of magnitude as for the simulated chains and suggests reasonable agreement between simulations and experiment. This both experimentally confirms previous work^11^, predicting the existence of an optimized region for signal propagation in anisotropy-engineered nanomagnet chains, and allows us to conclude that nanosecond current-generated field pulsing is an effective clocking mechanism that can be used in an ultrafast measurement to reset chains over millions of cycles. In addition, we predict (with micromagnetic simulations similar to Fig. 2g and Supplementary Fig. 3) that error-free signal propagation using nanosecond clocking pulses is extendable to even longer magnet chains (24 magnets) that incorporate biaxial anisotropy (Supplementary Note 3). This overcomes the error limitations exposed by exclusively using uniaxial magnets^9^.

### Dynamic magnetic imaging of signal propagation

To observe signal propagation in an anisotropy-engineered chain at nanosecond timescales, we use TR-PEEM. Notched nanomagnet chains with identical dimensions to the previous experiment (with lengths, *L*, from 300 to 500 nm) are fabricated on a Au wire on top of a Si wafer (Fig. 1b) and mounted to a customized circuit board designed to apply nanosecond current pulses (Fig. 1c, see Methods section). The 2-ns current pulses (which generate 100 mT on-chip fields) are synchronized with 70 ps X-ray pulses at a repetition rate of 3 MHz. The time delay between the current and X-ray pulses is varied with a pulse delay generator. XMCD contrast images (Fig. 1f) are generated by aligning and dividing PEEM images illuminated with left and right circularly polarized X-rays. As before, the contrast direction is along the nanomagnet easy axis in each chain. For a given delay, each image is an average of over 300 million clock cycles.

**Figure 3:**
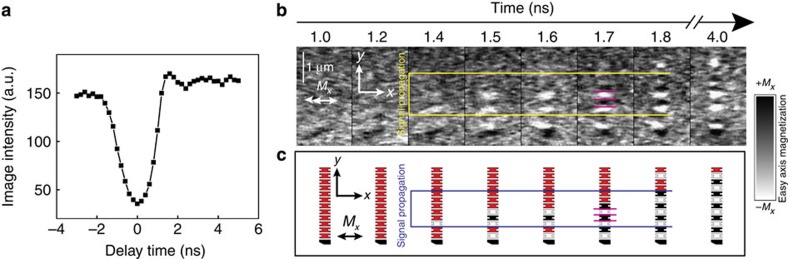
Observation of signal propagation in an NML chain by dipolar coupling. (**a**) Photoelectron intensity count for an image containing the Au wire versus time delay. (**b**) Averaged TR XMCD-PEEM images of a nanomagnet chain at various time delays from 1 to 4 ns compared with a 1-μm scale bar. The chain demonstrates behaviour that suggests dipolar coupling is switching magnets sequentially from the hard axis (along *ŷ*) to an antiparallel configuration (black and white contrast) on the easy axis (along 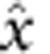) at fast timescales on the order of 100 ps. Signal propagation from the third nanomagnet to the eighth nanomagnet driven by dipolar coupling (emphasized in the yellow region) is observed between images 1.4 and 1.8 ns. Pink lines distinguish individual nanomagnets in the chain. (**c**) An interpretation of the switching events are observed in **b**. Red indicates magnets that are either in their metastable state after clocking or indistinguishable due to contrast greying. Black and white indicate magnets that have oriented along the easy axis 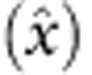 in opposite directions, respectively. Signal propagation from the third nanomagnet to the eighth nanomagnet driven by dipolar coupling (emphasized in the blue region) is observed between images 1.4 and 1.8 ns. Pink lines distinguish individual nanomagnets in the chain.

Figure 3a plots the integrated photoemission yield from a PEEM image of the wire versus time delay. The Lorentz force generated by the clock field deflects emitted photoelectrons during the clock pulse, shifting and reducing the intensity of the image. This confirms the generation of 2 ns magnetic field pulses and identifies the zero time delay point at the peak of the pulse. Figure 3b shows a series of TR XMCD-PEEM images of a chain with *L*=450 nm taken at delay times between 1 and 4 ns. Surface roughness and other lithographical irregularities may contribute to errors in this system^8^,^24^,^31^,^32^ while random signal propagation errors from individual clocking cycles are averaged into each image. This leads to contrast greying from individual nanomagnets that do not reproducibly reorient in the same direction each cycle. Figure 3c illustrates our interpretation of the signal propagation in this chain. All magnets marked in red are assumed to be either initialized along their hard axis or indistinguishable due to averaging. Black and white values indicate nanomagnets that reproducibly align to a single orientation. We also note that the images obtained near the falling edge of the current pulse (1.0–1.4 ns) were subject to electronic jitter causing image blurring making interpretation more difficult (this can clearly be seen in the evolving appearance of the first black input magnet).

As described in Supplementary Note 4, imaging at zero delay (Supplementary Fig. 4) confirms all nanomagnets in the chain are aligned along the hard axes (along **ŷ**); no contrast is observed during the clocking pulse since the magnetization vector of each magnet is perpendicular to the XMCD contrast direction (along the easy axis, 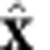). One nanosecond after the clocking pulse peak, the input magnet sets the initial condition for signal propagation; the remaining magnets remain aligned along their hard axis. At 1.4 ns, we observe the first repeatable switching of a nanomagnet; it appears to be an error that starts at the third nanomagnet of the chain. Despite this error, between 1.4 and 1.8 ns, we observe the sequential reorientation of nanomagnets starting from the third nanomagnet. The relevant magnets that reproducibly switch are boxed in yellow in Fig. 3b and blue in Fig. 3c. This confirms that signal propagation proceeds at a rate of ~100 ps per switching event, as predicted previously through computational NML studies^2^,^10^,^11^. In the 4-ns image, the signal propagation throughout the entire chain is complete and appears error free, however, the dynamics measurement has revealed both the error at the beginning of the chain and another near the end where a nanomagnet nucleated out of sequence in the 1.8-ns image.

The TR technique we have used introduces the capability of interrogating the performance of individual nanomagnets during signal propagation, a feature not present in existing quasi-static imaging measurements. Experimentally evaluating the performance of NML chains complements existing TR micromagnetic simulations (similar to the one demonstrated in Supplementary Fig. 3) offers realistic assessments of nanomagnet designs, and identifies systematic error nucleation sites and other architectural weaknesses resulting from environmental aberrations and lithographic limitations. In addition, this experimental observation verifies the fundamental mechanism upon which NML architectures are based. The sub-nanosecond regime of switching we have observed is governed by Landau–Lifshitz–Gilbert dynamics^10^ and is distinct from the typically slower thermally assisted switching^13^ that follows a modified Arrhenius model^19^.

### Single spin model to optimize reliability

**Figure 4:**
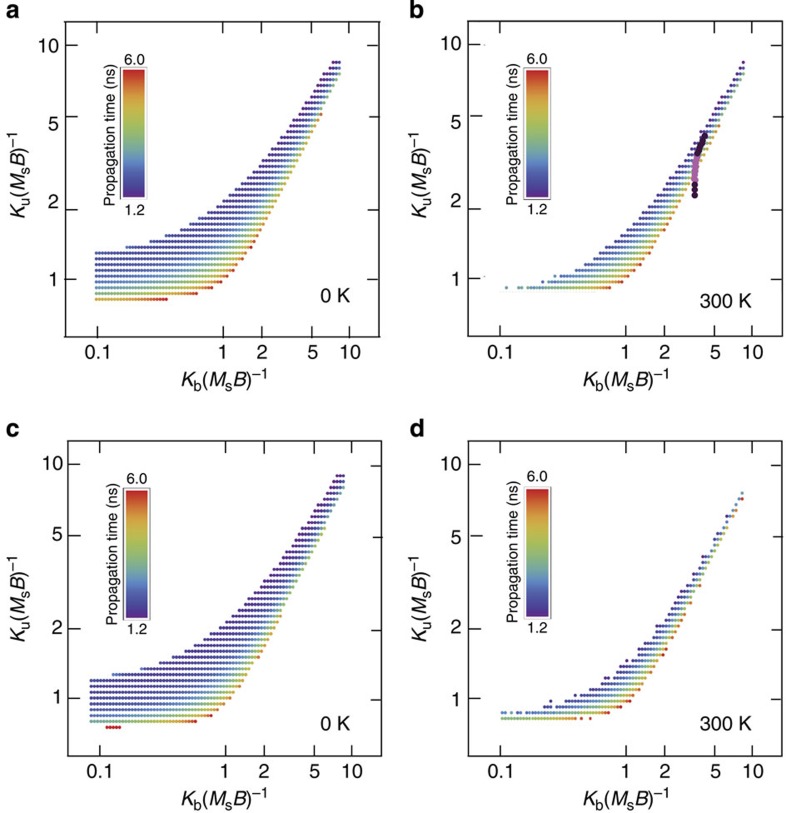
Reliability phase space calculated by macrospin simulations and comparison with micromagnetic simulations. (**a**,**b**) Log–log macrospin simulation plots for chains of 12 magnets (150 nm wide and 12 nm thick) separated by 30 nm at (**a**) 0 K and (**b**) 300 K that indicate values for *K*_u_ and *K*_b_, respectively (normalized to the product of the saturation magnetization, *M*_s_, and dipolar field coupling, *B*), which produce repeatable and reliable signal propagation starting from an ideally initialized metastable state. (**c**,**d**) Log–log macrospin simulation plots for chains of 12 magnets (50 nm wide and 12 nm thick) separated by 20 nm at (**c**) 0 K and (**d**) 300 K that indicate values for *K*_u_ and *K*_b_ (normalized to the product of the saturation magnetization, *M*_s_, and dipolar field coupling, *B*), which produce repeatable and reliable signal propagation starting from an ideally initialized metastable state. Simulations run at 300 K are performed 20 times per data point to statistically account for stochastic thermal effects. The colour scale indicates the average signal propagation time to complete each successful simulation. The anisotropy values from Fig. 2a and micromagnetic simulation signal propagation distance from Fig. 2g for ideal clocking at 300 K are overlaid onto **b**. The magenta colouring in these points indicates higher signal propagation distance.

To obtain a better physical understanding of the experimental data, we investigate both an adiabatic analytic model based on first-principles equations at 0 K (Supplementary Note 5) in addition to macrospin simulations that vary the temperature, nanomagnet size, anisotropy energy and dipolar coupling strength to identify parameter space requirements for perfect signal propagation. In the macrospin model, each magnet in the chain is represented by a single moment that possesses specific values for *K*_b_ and *K*_u_ that we independently vary over many simulations. Each chain is stabilized in the metastable state and simulations are run for both 0 and 300 K (at 300 K each simulation is performed 20 times). Both *K*_b_ and *K*_u_ are normalized by the dipolar coupling strength *M*_s_*B*, where *M*_s_ is the saturation magnetization and the dipolar coupling field 
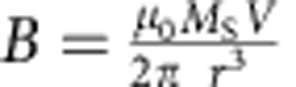
, where *μ*_0_ is the permeability of free space, *V* is the nanomagnet volume and *r* is the centre-to-centre magnet spacing. We perform simulations in a logarithmic grid of values between 0.1 and 10 for *K*_b_(*M*_s_*B*)^−1^ and *K*_u_(*M*_s_*B*)^−1^, and track signal propagation in each scenario. The simulations presented in Fig. 4 (on a log–log scale) represent the values for *K*_b_(*M*_s_*B*)^−1^ and *K*_u_(*M*_s_*B*)^−1^ that produced perfect, statistically significant and repeatable signal propagation for 150-nm-wide nanomagnets separated by 30 nm (Fig. 4a,b) and 50-nm-wide nanomagnets separated by 20 nm (Fig. 4c,d) at 0 and 300 K, respectively. The colour of each data point represents the average signal propagation time for each successful simulation that varies from 1.2 to 6 ns. Faster propagation times result when the anisotropy field of the easy axis increases with respect to the hard axis leading to faster magnet-to-magnet switching as expected from Gilbert damping. These simulated plots predict a phase space of reliable propagation based on the fundamental parameters of this system and agree well with a complementary plot analytically calculated from the adiabatic first-principles model (Supplementary Fig. 5).

At 0 K, the reliability phase space for both magnet sizes is similar (Fig. 4a,c). The most tolerant signal propagation regions that accept large proportional deviations in both *K*_b_ and *K*_u_ are found in the lower left of both plots, where the coupling fields are relatively strong. This suggests that good tolerance can be experimentally realized by choosing magnetic materials with a larger *M*_s_ and/or decreasing the spacing between nanomagnets. As the coupling strength in Fig. 4 is reduced (upper right in each plot), we observe less tolerance for perfect signal propagation. When we incorporate stochastic temperature effects into our simulations (Fig. 4b,d), we observe a reduction in the proportional tolerance for all magnet dimensions and coupling strengths. Thermal excitations increase the probability for errors to occur, diminishing the upper bound of the reliability phase space. In Fig. 4b, we overlay the anisotropy values and propagation distance probabilities for the ideal clocked, 300 K simulation data taken from Fig. 2a,g, which correspond to the energy range of the anisotropy-engineered magnets investigated in this work. We observe that this micromagnetic range crosses a relatively narrow region of successful signal propagation that compares well with both our OOMMF simulations and experimental observations of signal propagation reliability in Fig. 2. From an engineering perspective, the plots in Fig. 4 provide a guide for improving the performance of future NML architectures. Fabricating notched nanomagnets with values for *K*_u_(*M*_s_*B*)^−1^ and *K*_b_(*M*_s_*B*)^−1^ indicated by the blue data points optimizes the speed and reliability of signal propagation. While these simulations are motivated by the lithographically tunable nanomagnets, the findings from these anisotropy models are also applicable to magnets with magnetocrystalline anisotropy^10^,^11^. The fixed intrinsic biaxial properties of these materials combined with nanomagnet designs less susceptible to processing variations may provide a more scalable platform to optimize signal propagation in NML chains.

## Discussion

In summary, this work presents the first experimental evaluation of NML operation at its ultimate intrinsic speed. By clocking NML systems at high repetition rates with nanosecond pulses, we have performed experiments that both confirm the fundamental physical mechanism of NML technology and assess NML performance with computationally relevant cycling protocols. The dipolar coupling dynamics responsible for signal propagation in NML chains, which occurs at speeds near 100 ps per switching event, have been directly observed using TR magnetic X-ray microscopy. Our computational models strongly support our experimental evidence and provide a deeper insight that will help to engineer the reliability of NML systems, where the anisotropies and dipolar coupling strengths can be optimized by a judicious choice of the nanomagnet geometry, spacing and material.

## Methods

### Sample preparation for static magnetic measurements

Using electron beam lithography, electron beam evaporation and liftoff, chains of 12 nanomagnets (2 nm Ti/12 nm permalloy, 150 nm wide and 300–360 nm long), spaced apart by 30 nm, are produced on commercially available 500-μm-thick Si wafers capped on both sides with 100 nm layers of low-stress Si_3_N_4_. Each rectangular nanomagnet has two 50-nm-wide and 30-nm-deep notches centrally patterned along its width. The nanomagnet chains are bound on either end by shape-biased inputs^23^, which set the magnetic orientation of the first magnet in each chain, and blocks^8^, which stabilize the final magnet in each chain. Next, we patterned 6-μm-wide and 150-nm-thick aluminium wires capped with a 10-nm layer of copper on top of the chains using optical lithography and liftoff. Finally, to create a membrane for X-ray transmission, a lithographically defined window of the backside Si_3_N_4_ layer and Si substrate is etched away releasing the remaining Si_3_N_4_ layer with the existing nanomagnet and wire features. The resistance of the Al wire after processing is 21 Ω. The membrane samples are then mounted on a Rogers 4350B printed circuit board and electrically contacted with conductive silver paint.

### Transmission X-ray experimental setup

We used the full-field transmission soft X-ray microscope (XM-1 at beamline 6.1.2) at the ALS at Lawrence Berkeley National Laboratory to obtain magnetic contrast images of our nanomagnet chains. Samples are mounted in ambient conditions at room temperature with the surface normal tilted 30° to the X-ray optical pathway to obtain a projection of the sample magnetization onto the photon propagation direction. XMCD at the Fe L_3_ absorption edge (707 eV), that is, magnetization-dependent absorption of circularly polarized X-rays transmitting through the sample, provides magnetic contrast. To enhance the magnetic contrast, each image is divided on a pixel-by-pixel basis by a reference image that is recorded in an external magnetic field strong enough to saturate the nanomagnets along their easy axes. To observe nanomagnet propagation statistics, MTXM images are repeatedly recorded after manually triggering single-clocking pulses along the Al wire. Each pulse is 3 ns long with an amplitude of 18.5 V. This generates an 880-mA current that produces an on-chip magnetic field of ~84 mT.

### Sample preparation for dynamic magnetic measurements

Nanomagnet chains are fabricated on silicon wafers coated with a 100-nm-thick insulating layer of SiO_2_. A gold metal strip, 6-μm wide and 160-nm thick with 2 nm Ti adhesion layer is patterned on the surface using photolithography, thermal evaporation and liftoff. A 45-nm-thick layer of spin-on aluminium oxide phosphate (Inpria Corp.) was deposited on the sample in order to smooth the surface of the Au wire on which the nanomagnet chains are fabricated. Next, chains of 12 nanomagnets (2 nm Ti/12 nm permalloy/2 nm Al) bounded by shape-biased inputs^23^ and blocks^8^ are patterned by electron beam lithography. The nanomagnets in each chain are 150 nm wide with 30 nm gaps between them, and vary in length from 300 to 500 nm. Each rectangular nanomagnet has two 50-nm-wide and 30-nm-deep notches centrally patterned along its long axis edges. A final 1 nm film of platinum is sputtered over the entire sample surface to reduce surface charging during the PEEM measurement. The resistance of the Au wire after processing is 7 Ω.

### TR X-ray experimental setup

Our dynamics experiment is set up as a stroboscopic pump-probe measurement where fast current pulses along the strip are synchronized with X-ray pulses from the ALS at beamline 11.0.1 during the two-bunch operation mode. We designed and built a customized sample holder to generate the current pulses while isolating the critical electronic components from the PEEM high-voltage electron optics. The requirement in the PEEM instrument to bias the entire sample holder at high voltage (12–18 kV) and the need for sub-nanosecond rise and fall times dictated the use of an optical link in place of electrical feedthroughs to trigger the current pulse. The *in situ* pulser circuit was built from commercially available surface mount components, an avalanche photodiode (APD) and a polyimide printed circuit board, with a silver-plated copper conductor. When the APD is irradiated by a short, infrared laser pulse, it produces an electrical pulse that is amplified by two stages of radio frequency amplifiers. Current pulses with a peak amplitude of up to 1 A are delivered into the Au strip by this circuit. A near field calculation using the superposition integral formulation of the Biot–Savart law estimates that the amplified pulses produce an in-plane magnetic field with a peak amplitude of 100 mT at the location of the chains on the surface of the strip. This field is our clock mechanism (Supplementary Fig. 4). The sample and circuit assembly contacts a copper heatsink to dissipate heat from both amplifiers. We nominally operate this measurement at room temperature inside the PEEM vacuum chamber. The probe beam consists of 70 ps X-ray pulses at a repetition rate of 3 MHz. Pulses (850 nm) with 1–2 ns duration from a fast diode laser strike the APD at the same repetition rate. The diode laser is mounted outside the PEEM vacuum chamber and the beam is free-space coupled through a window port and focused onto the APD. A pulse delay generator triggered by a synch pulse from the synchrotron timing system synchronizes the X-ray bunches to the laser pulse. Accumulating magnetic contrast images in the PEEM by varying the delay allows us to study the time dynamics.

As with the MTXM images, we obtain magnetic contrast by XMCD tuning the X-rays to the Fe L_3_ absorption edge, expose two images at the same location illuminated by right and left circularly polarized X-rays, respectively, and then compare them by per-pixel numerical division. The images are then adjusted using a median noise filter and linear brightness and contrast stretching calibrated with nearby non-magnetic regions. We imaged 22 chains and each image is averaged over 360 million clock cycles (2 min integration time).

## Author contributions

MTXM samples were prepared by Z.G., D.B.C. and W.C. MTXM measurements were performed by Z.G., M.-Y.I. and P.F. TR-PEEM samples were prepared by Z.G., D.B.C., W.C. and P.B. TR-PEEM experiments were performed by Z.G., M.E.N., R.S., J.H., B.L., M.T.A., M.A.M., A.D., A.Y. and A.S. Simulations were performed by Z.G. and B.L. The experiments were conceived by J.B. and the manuscript was written by M.E.N., Z.G. and J.B. All authors discussed the results and commented on the manuscript.

## Additional information

**How to cite this article:** Gu, Z. *et al*. Sub-nanosecond signal propagation in anisotropy-engineered nanomagnetic logic chains. *Nat. Commun*. 6:6466 doi: 10.1038/ncomms7466 (2015).

## Supplementary Material

Supplementary Information Supplementary Figures 1-5, Supplementary Notes 1-5, and Supplementary References.
