# Supplementary material for: Sub-nanosecond signal propagation in anisotropy-engineered nanomagnetic logic chains
Source: Nat Commun. 2015 Mar 16;6:6466. doi: 10.1038/ncomms7466 (PMC4382687; doi:10.1038/ncomms7466)
Supplement: Supplementary Information — Supplementary Figures 1-5, Supplementary Notes 1-5, and Supplementary References. [file ncomms7466-s1.pdf]

Supplementary Figure 1

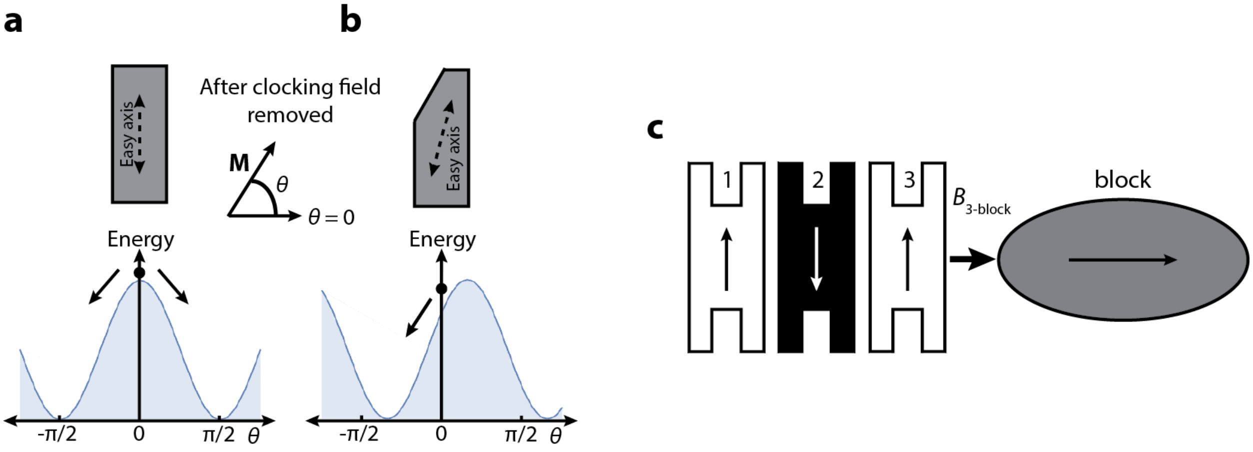

Supplementary Figure 1: Design of input and block magnets. a) Schematic of an input nanomagnet without engineered shape anisotropy. The corresponding energy diagram plots the magnetic energy potential as a function of the magnetization orientation (**M**) in the nanomagnet with respect to the hard axis (θ = 0). In the case with no engineered shape anisotropy a nanomagnet initialized along its hard axis (position of the black dot) has an equal probability of switching into either state (up or down) along the easy axis. b) Schematic of an input nanomagnet with engineered shape anisotropy. The corresponding energy diagram plots the magnetic energy potential as a function of the magnetization orientation in the nanomagnet with respect to the hard axis (θ = 0). In the case with engineered shape anisotropy a nanomagnet initialized along its hard axis (position of the black dot) favors switching to only one direction along the easy axis. c) A chain of nanomagnets are terminated by an ellipse-shaped block nanomagnet which applies a dipolar coupling field $B_{3\text{-block}}$ on the final nanomagnet in the chain.

Supplementary Figure 2

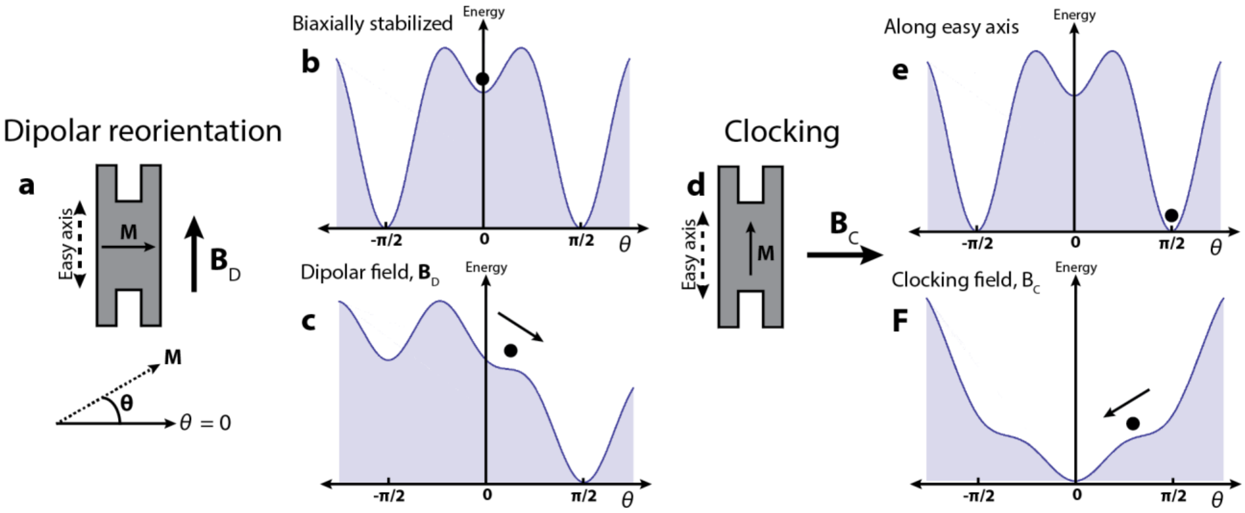

Supplementary Figure 2: Magnetization rotation due to the clocking and dipolar fields. a) Schematic for a nanomagnet initialized along its hard axis (θ = 0) and stabilized by biaxial anisotropy being influenced to rotate by a dipolar coupling field ($\mathbf{B}_D$) from a neighboring nanomagnet. The magnetic energy potential as a function of the magnetization orientation (indicated by the black dot) of the nanomagnet with respect to the hard axis for a nanomagnet biaxially stabilized along its hard axis is plotted (b) before and (c) after the influence the dipolar field. At a critical dipolar field value (B_D) the magnetization of the nanomagnet is driven to rotate from the hard axis to the easy axis. d) Schematic for an uninitialized nanomagnet oriented along its easy axis (θ = π/2) being initialized by a clocking field ($\mathbf{B}_C$). The magnetic energy potential as a function of the magnetization orientation (indicated by the black dot) of the nanomagnet with respect to the hard axis for an uninitialized nanomagnet is plotted (e) before and (f) after the influence a clocking field. At a critical clocking field value (B_C) the magnetization of the nanomagnet is driven to rotate from the easy axis to the hard axis.

Supplementary Figure 3

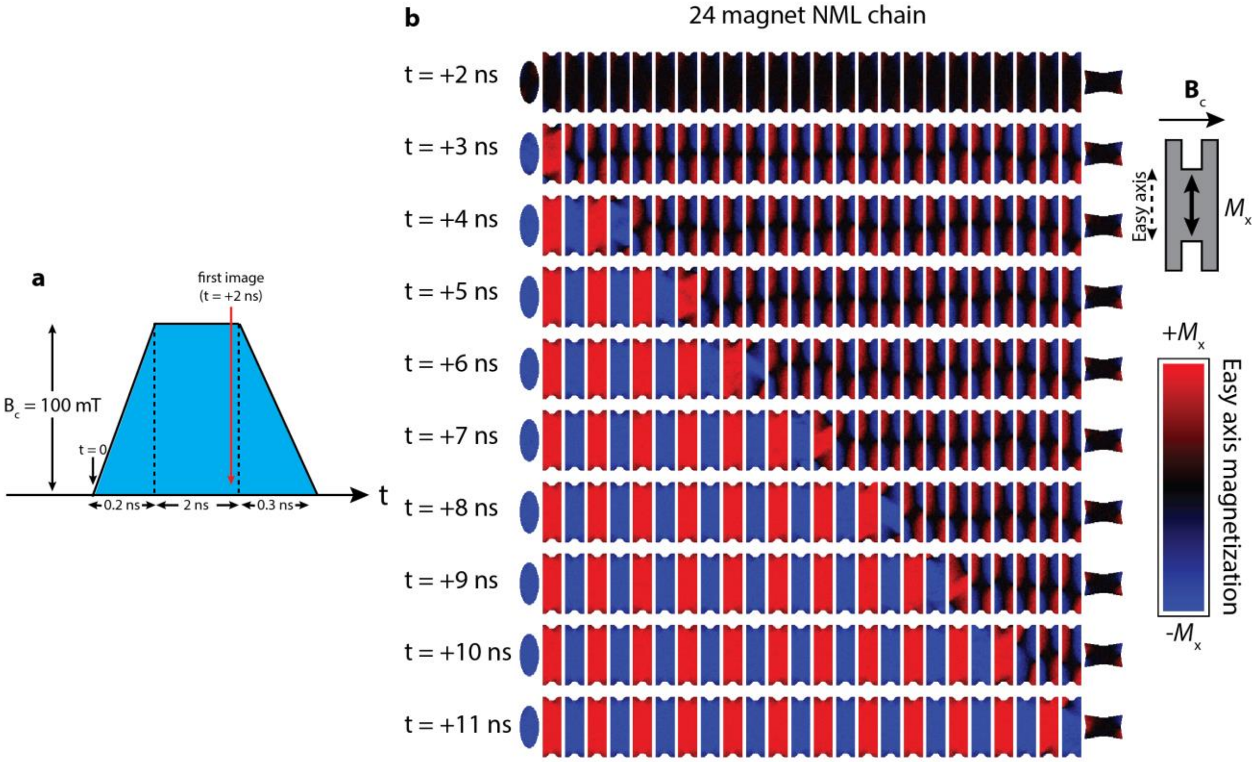

Supplementary Figure 3: OOMMF simulation of signal propagation in longer chains. a) Clocking pulse profile applied prior to behavior observed in part b). b) Perfect signal propagation in a chain with 24 nanomagnets with engineered biaxial anisotropy, initialized by a clocking field (B_c = 100 mT) applied perpendicular to the nanomagnet easy axis, simulated at room temperature. The red and blue colors correspond to the up ($+M_x$) and down ($-M_x$) state along the easy axis as shown in the inset.

Supplementary Figure 4

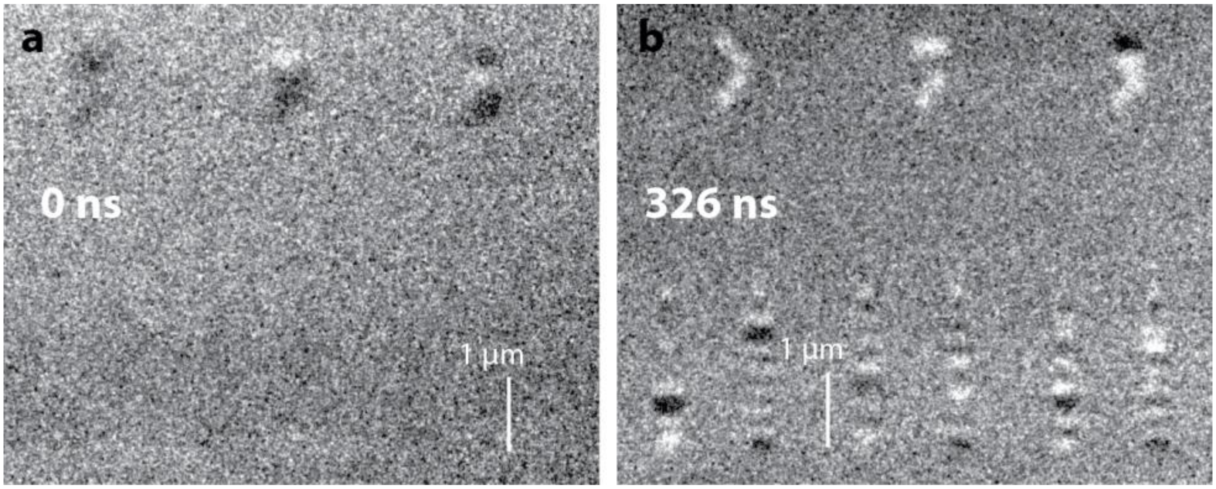

Supplementary Figure 4: Magnetic contrast images indicate successful pulse clocking. a) XMCD-PEEM image taken at 0 ns delay time. Index magnets show magnetic contrast. Magnets along the wire are oriented along their hard axis and are perpendicular to the magnetic contrast direction (along the easy axis). b) XMCD-PEEM image taken at 326 ns delay time. Index magnets show magnetic contrast. Magnets along the wire are oriented along their easy axis and are parallel to the magnetic contrast direction.

Supplementary Figure 5

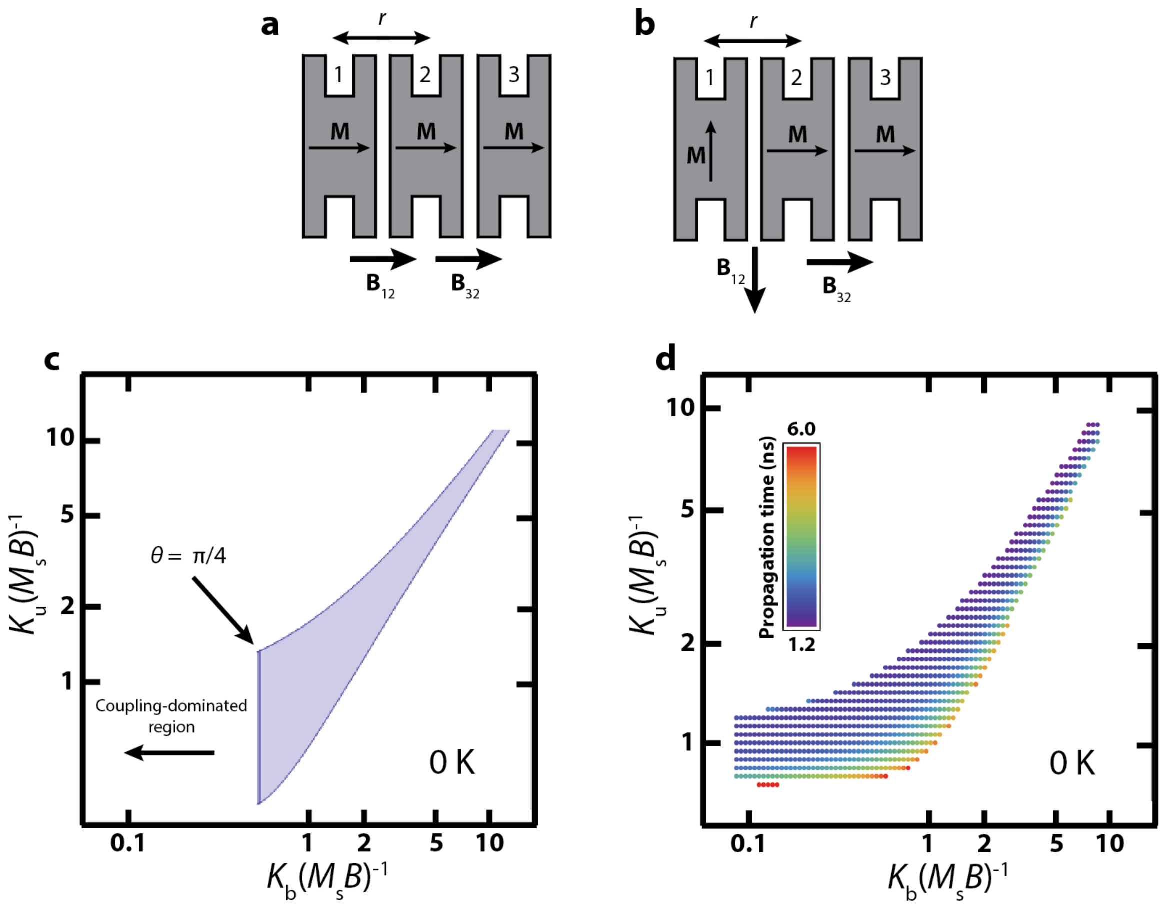

Supplementary Figure 5: Calculating clocking stability and propagation reliability analytically. a) Schematic of the magnetic orientation for 3 nanomagnets stabilized along their hard axis. Magnet 1 and 3 act on magnet 2 via their dipolar fields ($\mathbf{B_{12}}$ and $\mathbf{B}_{32}$) which are parallel to the magnetization in the metastable state. b) Schematic of the magnetic orientation for 3 nanomagnets during signal propagation, where the signal is propagating from magnet 1 to 3. The dipolar field $\mathbf{B}_{12}$ is now oriented perpendicular to the metastable magnetization state of magnet 2. c) Analytical calculation, performed at 0 K, of a reliable propagation region based on the geometry of part b) plotted on a log-log scale as a function of K_b, K_u, M_s, and the dipolar coupling, B. A coupling-dominant region is indicated, but not plotted (see Supplementary Note 5). d) Macro-spin simulation reliability plot for a 12 magnet chain with 50 nm wide, 12 nm thick nanomagnets separated by 30 nm at 0 K plotted on a log-log scale as a function of K_b, K_u, M_s, and the dipolar coupling, B. The coloring indicates the total calculated time required to propagate signal in the chains with 100% reliability. The coupling-dominant region is predicted and plotted.

Supplementary Note 1

Input magnets

Ensuring repeatable performance in our magnet chains for the stroboscopic time-resolved measurement was critical. We required the behavior of each individual magnet to be identical each clock cycle. To accomplish this, the easy axis of each input magnet was rotated with shape anisotropy engineering such that a component of the easy axis was projected along the clocking field direction. After the clocking field is removed an input magnet with shape anisotropy (Supplementary Figure 1b) strongly prefers to relax into only one easy axis state; for contrast an input magnet without shape anisotropy (Supplementary Figure 1a) would have an equal probability of relaxing into either degenerate easy axis state[1].

Terminating block magnets

The final magnet in the chain requires a special terminating nanomagnet neighbor called a block. In the absence of this block a final magnet with only one neighbor is susceptible to relaxing out-of-sequence due to an absence of symmetric hard axis coupling fields which typically provide stability in magnets with two neighbors (Supplementary Note 5). To add stability to the final magnet, an ellipse-shaped block magnet with an easy axis parallel to the clocking field direction is fabricated next to the final magnet to provide an additional coupling field $\mathbf{B}_{3\text{-block}}$ as shown in Supplementary Figure 1c[2].

Supplementary Note 2

Calculating K_b and K_u using an analytical model and OOMMF

The values of the biaxial (K_b) and uniaxial (K_u) anisotropy energy in Figure 2a of the main text are estimated using both an analytical model and micromagnetic simulations. Using an analytical model, we first calculate the relationship between the magnetic dipolar coupling field (B_D) and the clocking field (B_C) as a function of K_b, K_u, and the magnetization saturation (M_S). To determine these functions we account for the critical fields (dipolar and clocking) required to rotate the nanomagnet magnetization between its easy and hard axes in two cases. In the first case, we consider a nanomagnet biaxially stabilized along its hard axis under the influence of a dipolar coupling field from a neighboring magnet (B_1) applied perpendicular to the magnetization as shown in Supplementary Figure 2a. In the second

case, we consider the influence of an external clocking field (B_2) applied along the nanomagnet hard axis on an uninitialized nanomagnet stabilized along its easy axis as shown in Supplementary Figure 2d.

The energy equation describing the first scenario (Supplementary Figure 2a) is

$$U = -K_u \sin^2\theta - \frac{K_b}{4}\cos^2 2\theta - M_S B \sin\theta \quad (1).$$

Assuming $K_b > K_u$ the moment can be stabilized in a metastable region along the hard axis as shown in Supplementary Figure 2b, where $\theta = 0$ corresponds to the hard axis. Applying a dipolar field B_1 tilts the energy landscape until the energy barrier defined by K_b and K_u becomes a saddle point at $\frac{dU}{d\theta} = 0$ and $\frac{d^2U}{d\theta^2} = 0$ (Supplementary Figure 2c). Solving these equations for B_1 in the first quadrant gives

$$B_D = \frac{4}{3M_S}\sqrt{\frac{(K_b - K_u)^3}{6K_b}} \quad (2),$$

where B_D is the value of B_1 at the saddle point. In this case $K_b \geq K_u$ is required to obtain a real solution, otherwise no saddle point emerges since there is no energy barrier.

The energy equation describing the second scenario (Supplementary Figure 2d) is

$$U = -K_u \cos^2\theta - \frac{K_b}{4}\cos^2 2\theta - M_S B \sin\theta \quad (3).$$

The moment starts stabilized along the easy axis as shown in Supplementary Figure 2e, where $\theta = 0$ corresponds to the hard axis. Applying a clocking field B_2 tilts the energy landscape until the energy barrier defined by K_b and K_u becomes a saddle point at $\frac{dU}{d\theta} = 0$ and $\frac{d^2U}{d\theta^2} = 0$ (Supplementary Figure 2f). Solving these equations for B_2 in the first quadrant gives

$$B_C = \frac{4}{3M_S}\sqrt{\frac{(K_b + K_u)^3}{6K_b}} \quad (4),$$

where B_C is the value of B_2 at the saddle point. In this case there is no requirement of $K_b \geq K_u$ because there is always an energy barrier going from the easy axis to the hard axis due to K_u.

We calculate B_D and B_C for the nanomagnet geometries used in our experiments using micromagnetic simulations in OOMMF[3]. Within the geometries simulated, the largest B_C was 45 mT, meaning that clocking fields exceeding 45 mT by a reasonable margin are sufficient to initialize the nanomagnet chains. K_b and K_u for each geometry are calculated using equations 2 and 4. This assumes the energy barrier defined by K_b and K_u can be traversed from both the easy axis and metastable hard axis. This biaxial anisotropy approximation addresses the essential elements of NML: determining the field energy

required via dipolar coupling and external clocking to reorient moments between the easy and hard axes over many cycles.

Supplementary Note 3

To demonstrate the superior stability gained by engineering a metastable state with biaxial anisotropy, we simulate signal propagation in a chain twice as long as the chains we measured with time-resolved photoemission electron microscopy (TR-PEEM). Each magnet (excluding the input and the block) in the 24 magnet chain of Supplementary Figure 3b is configured with dimensions identical to those used in the TR-PEEM experiment: 450 nm x 150 nm and 12 nm thick. The OOMMF simulation[3] (at T = 300 K) is initialized with a trapezoidal-shaped 2 ns clocking field pulse (B_c) of 100 mT (Supplementary Figure 3a) oriented along the hard axes of the nanomagnets. This pulse is similar to the one used in the simulations of Figure 2g of the main article. After the clocking pulse is removed we confirm each individual nanomagnet remains stability oriented along its hard axis until it is excited by its left-most nearest neighbor. This simulation, which depicts perfect signal propagation in ambient conditions, demonstrates that with a judicious choice of biaxial anisotropy accurate propagation is achievable in chains of arbitrary lengths.

Supplementary Note 4

During our time-resolved PEEM measurement we vary the delay time between the clocking pulse and the x-ray pulse with a delay generator. Figure 3a in the main manuscript characterizes the clocking pulse by measuring the photo-electron intensity of a PEEM image as a function of delay time. During the rising and falling edges images appear blurry and move due to the Lorentz force acting on the electrons and the time averaging of jitter. However at the peak of the pulse, images are stable and we observe magnetic contrast of index magnets fabricated off of the wire designed to indicate the specific nanomagnet length in each chain (Supplementary Figure 4a). Along the wire we observe no magnetic contrast which indicates that during the pulse all nanomagnets are aligned along their hard axis, perpendicular to the XMCD contrast direction which is parallel to the nanomagnet easy axis. In Supplementary Figure 4, we compare an image taken at the pulse peak (Supplementary Figure 4a) with an image taken at 326 ns after the pulse peak (Supplementary Figure 4b). We also observe that the pulse field can influence the orientation of the indexing magnets off the wire.

Supplementary Note 5

Clocking stability

To analytically derive a condition for the clocking stability we consider three closely spaced magnets (Supplementary Figure 5a) with both uniaxial (K_u) and biaxial anisotropy (K_b) separated by a center-to-center distance, *r*, and calculate the influence of dipolar fields ($\mathbf{B}_{12}$ and $\mathbf{B}_{32}$) from the edge magnets (1 and 3) onto the central magnet (2). Assuming the magnets are initialized by an external clocking field orienting the magnets along their hard axes, we calculate the maximum value of K_u for the three magnets, such that magnet 2 remains in the metastable state.

The total energy of magnet 2, including both uniaxial and biaxial anisotropy terms and the dipolar fields from both neighboring magnets is

$$U = -K_u \sin^2\theta - \frac{K_b}{4}\cos^2 2\theta - 2M_S B\cos\theta \quad (5),$$

where B is the dipolar coupling field from one neighboring magnet ($B = B_{12} = B_{32}$),

$$B = \frac{\mu_0}{4\pi}\left(\frac{3\vec{r}\cdot(\vec{m}\cdot\vec{r})}{r^5} - \frac{\vec{m}}{r^3}\right) = \frac{\mu_0 M_S V}{2\pi r^3} \quad (6),$$

given $\vec{r}\cdot(\vec{m}\cdot\vec{r}) = mr^2$ for a nanomagnet volume *V*. For magnet 2 to remain magnetized along its hard axis there must be an energy barrier defined by K_b and K_u. If K_u is raised with respect to K_b the energy barrier is reduced. At $\frac{dU}{d\theta} = 0$ and $\frac{d^2U}{d\theta^2} = 0$ the energy barrier becomes a saddle point which allows spontaneous realignment from the hard axis to the easy axis. Solving these equations gives

$$K_u < K_b + M_S B \quad (7)$$

which defines conditional bounds for K_u and K_b with respect to *B* for stable clocking into the metastable state.

Transmission reliability

To analytically derive a condition for the propagation reliability we consider three closely spaced magnets (Supplementary Figure 5b) with identical parameters as above, however now we assume signal propagation is occurring (after stable clocking) from magnet 1, which has been reoriented along its easy axis. Again, we calculate the influence of the dipolar fields ($\mathbf{B}_{12}$ and $\mathbf{B}_{32}$) from the edge magnets (1 and 3) onto the central magnet (2) to calculate the parameter requirements necessary for dipolar field realignment in magnet 2.

Because B_{12} and B_{32} are no longer equal:

$$B_{12} = \frac{\mu_0}{4\pi}\left(\frac{3\vec{r}\cdot(\vec{m}\cdot\vec{r})}{r^5} - \frac{\vec{m}}{r^3}\right) = \frac{\mu_0 M_S V}{2\pi r^3} \quad (8),$$

given $\vec{r}\cdot\ (\vec{m}\cdot\vec{r}) = mr^2$ and

$$B_{32} = \frac{\mu_0}{4\pi}\left(\frac{3\vec{r}\cdot(\vec{m}\cdot\vec{r})}{r^5} - \frac{\vec{m}}{r^3}\right) = -\frac{\mu_0 M_S V}{4\pi r^3} \quad (9),$$

given $\vec{m}\cdot\vec{r} = 0$, the total energy equation is now given by

$$U = -K_u \sin^2\theta - \frac{K_b}{4}\cos^2 2\theta - M_S B\left(\cos\theta + \frac{\sin\theta}{2}\right) \quad (10).$$

For magnet 2 to reorient, the energy barrier must be removed by the dipole fields from its neighbors. This occurs at a saddle point in the energy equation when $\frac{dU}{d\theta} = 0$ and $\frac{d^2U}{d\theta^2} = 0$ between $0 < \theta \leq 0.228\pi$. Solving these equations gives two expressions which define a region for propagation reliability in this system:

$$\frac{8K_b}{M_S B} < \frac{1}{2\sin^3\theta} + \frac{1}{\cos^3\theta} \quad (11)$$

and

$$\frac{8K_u}{M_S B} > \frac{1}{2\sin^3\theta} - \frac{1}{\cos^3\theta} - \frac{3}{\sin\theta} + \frac{6}{\cos\theta} \quad (12).$$

Within this region, propagation along a chain behaves as expected: magnet 2 will remain in the metastable state until it is driven by the dipolar fields of magnet 1 to its easy axis. We note that this model is adiabatic and is only valid at 0 K.

We also note that our calculation limitation of $0 < \theta \leq 0.228\pi$ suggests that there is no threshold past $\theta \approx 0.228\pi$. This is because $\frac{1}{2\sin^3\theta} + \frac{1}{\cos^3\theta}$ reaches its minimum value at $\theta \approx 0.228\pi$, however it is possible to choose smaller values for K_b, but there will be no saddle point, despite this magnetic reorientation is still permitted because a local energy minimum is present. Due to the small values of K_b relative to M_sB, we call this region coupling-dominated, where biaxial anisotropy is weak compared to dipolar coupling and no energy barrier impedes switching. Plotting equations 7, 11, and 12 illustrates a region of permissible parameters (K_b, K_u, M_s, and B) for reliable transmission (Supplementary Figure 5c). We compare this analytical plot to a plot generated using a macro-spin model similar to those plotted in Figure 4 of the main text (Supplementary Figure 5d) which calculated stable propagation in chains of 12 closely spaced nanomagnets. The coupling-dominant region is apparent in the macro-spin simulation.

The qualitative and quantitative nature of Supplementary Figure 5c and 5d improve the confidence of our macro-spin simulations. Our analytical model is two-dimensional, adiabatic, and is performed at 0 K. The simulations assume three-dimensional properties, time-dependent dynamics based on the Landau-Lifshitz-Gilbert (LLG) equation, and are performed at 0 and 300 K. Comparing these two simulations suggests a physically consistent understanding (even with a single macro-spin approximation) of the fundamental properties and performance of this system. Based on this understanding we are able to learn more about potential error mechanisms (e.g. non-nearest neighbor dipolar coupling which cause magnets two or more positions distant to reorient, or the effects of thermal fluctuations) and can make better design choices in future attempts to demonstrate improved reliability.

Supplementary References

1. Niemier, M.T., et al. Shape engineering for controlled switching with nanomagnet logic. *IEEE Trans. Nano.* **11**, 220-230 (2012).
2. Alam, M.T., et al. On-chip clocking of nanomagnet logic lines and gates. *IEEE Trans. Nano.* **11**, 273-286 (2012).
3. Donahue, M. J. & Porter, *D. G. OOMMF User's Guide, Version 1.0, Interagency Report NISTIR* 6376 (1999).
